# Root Medication

**Published:** 1888-04

**Authors:** S. Eschelman

**Affiliations:** Buffalo, N. Y.


ARTICLE III.

### ROOT MEDICATION.

BY S. ESCHELMAN, M. D., D. D. S., L. D. S., BUFFALO, N. Y.

[Read before the Buffalo Dental Society, December, 1887.]

Before considering medicaments used to destroy the septic products due to the putrefaction of the dental pulp, which, if not destroyed, produce, by mechanical irritation or septic infection, inflammatory action in the highly vascular tissues which constitute the root membrane, it will be well to consider some of the conditions which make it imperative to treat a putrescent pulp chamber.

Writers usually mention only one point of egress to the peridental membrane for septic matter. I believe such a statement to be faulty, for two reasons: 1st—if such were the case, the mere hermetical closing of the apical foramen would be sufficient to protect the vascular structures surrounding the root; 2d—clinical experience teaches us that such treatment soon receives a vigorous protest from the peridental membrane. Now, this being the case, let us see if the microscopic anatomy of the tooth does not show us another way of access to the peridental membrane. If we direct our attention to the manner in which the dentinal tubuli terminate, we find that they terminate in three ways: 1st—in loops; 2d—with the spaces of the granular layer; 3d—with the canaliculi of the cementum. We have, therefore, a direct continuation of living matter from the pulp to the peridental membrane, through the dentinal fibres connecting with the cells which occupy the lacunæ and canaliculi of the cementum.

Stowell believes that the dentine and cementum have a regular lymph canalicular system; corresponding in a general way, with the lymph canalicular system of the whole body. He says that neither the dentinal fibres in the dentinal tubuli nor the cells in the lacunæ and canaliculi of the cementum, nor the protoplasmic masses in the interglobular spaces, quite fill the space allotted to them, but leave room for the flow of lymph about them. By admitting that lymph spaces exist in the dentine and cementum, we can easily understand how septic matter,—due to putrefaction,—that is so injurious to the integrity of the peridental membrane, can enter; also why such agents as arsenic will destroy the pulp, when applied to the tooth with a thick layer of intervening dentine.

As to the medicaments which are chiefly used in placing the pulp chamber and its environments in an aseptic condition, I will consider their therapeutic value in meeting the three pathological conditions found in a putrescent pulp chamber; namely, 1st—their power of destroying the products of putrefaction; 2d—their power of destroying the agents of putrefaction, fungi; 3d—their power of coagulating or incorporating themselves with the dead remains of the dentinal fibres.

Regarding the products of putrefaction; sulphuretted hydrogen gas is the only one with which we will have to deal, and we find that the medicaments which act as true deodorants are those that contain chlorine, iodine, bromine, and oxygen, and some of the metallic salts; all of which decompose the sulphuretted hydrogen, forming solid or liquid compounds with it. Deodorants containing metals or bromine are not applicable as disinfectants of the pulp chamber. Only those containing chlorine, iodine and oxygen are useful.

In considering iodoform we include iodine also, as we know that it parts readily with its iodine when in solution, or when exposed to the sun or daylight in the presence of fats, by the application of heat or oxidizing agents, or in contact with blood. Nascent iodine decomposes sulphuretted hydrogen by uniting with the hydrogen, forming hydriodic acid, the sulphur being precipitated. Bichloride of mercury is another medicament which will decompose sulphuretted hydrogen, forming the black sulphide of mercury and hydro-chloric acid.

In peroxide of hydrogen we probably have our best and most elegant disinfectant. It is a very unstable compound, readily yielding its extra atom of oxygen, which proves destructive to sulphuretted hydrogen gas by its union with the sulphur, forming dioxide, and the elimination of free hydrogen gas. Carbolic acid, creosote, eucalyptus oil and the essential oils have no effects as true deodorants, but accomplish the object, if an opening is left for the escape of sulphuretted hydrogen gas, by virtue of their therapeutic value as medicaments, thus remedying the second pathological condition mentioned, namely, the presence of the agents of putrefaction, fungi. All of the deodorants mentioned, with the exception of peroxide of hydrogen, are antiseptics which stand at the head of their class.

After complete disinfection and sterilization of the pulp chamber and its environments, we come to the consideration of those medicaments which meet the third pathological condition mentioned, namely, those which coagulate or incorporate themselves with the dead albumenoid matter contained in the dentinal tubuli. After having established an aseptic condition, I believe it to be very important to use remedies which will keep the dead matter in the tubuli in an aseptic state, and antiseptics that form insoluble compounds with the dead matter by coagulating the albumen are the remedies to be selected.

Some have objected to the use of remedies that coagulate albumen, on the supposition that the pulpal end of the tubuli becomes closed and does not allow the medicament to penetrate to the terminal ends of the tubuli. Now this is a misstatement, which can easily be demonstrated by coloring carbolic acid with carmine and keeping the pulp chamber of a tooth filled with the acid for a few days; when it can readily be seen that it has permeated the tubuli to their terminal ends.

If we were to reject the medicaments which coagulate albumen we would be obliged to reject nearly all of our best antiseptics; as bichloride of mercury, nitrate of silver, carbolic acid, and chloride of zinc, all coagulate albumen. Iodine and iodoform will not coagulate albumen, but will incorporate themselves with it. Iodoform, while it is one of our best remedial agents in the conditions mentioned, possesses another valuable property in common with carbolic acid that the other medicaments mentioned do not possess: namely, anæsthetic properties, which, in my judgment, give it precedence in the conditions mentioned when complicated with vascular disturbance of the peridental membrane.—*The Dental Advertiser.*

